# Death of Jno. Murphy, M.D.

**Published:** 1870-05

**Authors:** 


***Obituary.***

We regret to notice the death, at Menominee, Mich., (March 11th ult.,) of John Murphy, M. D., a graduate of the class, '60

and '61, Rush Med. College. Dr. Murphy enlisted as a private soldier in the 37th Illinois Infantry, but was speedily promoted through the different grades to the Surgeoncy of a corps under General Banks. He served through the entire war, and at its close retired with a brilliant record, but unfortunately, like too many others, with a shattered constitution. Engaging in general practice, he shortly gained general confidence and esteem. The local paper comes to us with its columns dressed in mourning, and an account of the funeral, as conducted by the Masonic fraternity. Every evidence of sincere sorrow was manifested. Peace to his ashes, and honor to his memory.

